# CD105 positive neovessels are prevalent in early stage carotid lesions, and correlate with the grade in more advanced carotid and coronary plaques

**DOI:** 10.1186/2040-2384-1-6

**Published:** 2009-09-21

**Authors:** Ana Luque, Mark Slevin, Marta M Turu, Oriol Juan-Babot, Lina Badimon, Jerzy Krupinski

**Affiliations:** 1 Department of Neurology, Stroke Unit, Hospital Universitari Mutua Terrassa, Terrassa, Barcelona, Spain; 2 Cardiovascular Research Center, CSIC-ICCC, Hospital de la Santa Creu i Sant Pau (UAB), Barcelona, Spain; 3 Fundacio IDIBELL, Barcelona, Spain; 4 School of Biology, Chemistry and Health Science, Manchester Metropolitan University, Manchester, UK; 5 Faculty of Medical and Human Sciences, University of Manchester, Manchester, UK

## Abstract

**Background:**

Previous studies have demonstrated that expression of CD105 is a sensitive marker and indicator of endothelial cell/microvessel activation and proliferation in aggressive solid tumour growth and atherosclerotic plaque lesions. Since intimal neovascularization contributes significantly to subsequent plaque instability, haemorrhage and rupture.

**Methods:**

We have used immunohistochemical analysis to investigate the expression of CD105-positive vessels in both large (carotid) and medium calibre (coronary and middle cerebral artery, MCAs) diseased vessels in an attempt to identify any correlation with plaque growth, stage and complication/type.

**Results:**

Here we show, that carotid arteries expressed intimal neovascularization associated with CD105-positive endothelial cells, concomitant with increased inflammation in early stage lesions, preatheroma (I-III) whilst they were not present in coronary plaques of the same grade. Some of these CD105-positive neovessels were immature, thin walled and without smooth muscle cell coverage making them more prone to haemorrhage and rupture. In high-grade lesions, neovessel proliferation was similar in both arterial types and significantly higher numbers of CD105-positive *vasa vasorum *were associated with plaque regions in coronary arteries. In contrast, although the MCAs exhibited expanded intimas and established plaques, there were very few CD105 positive neovessels.

**Conclusion:**

Our results show that CD105 is a useful marker of angiogenesis within adventitial and intimal vessels and suggest the existence of significant differences in the pathological development of atherosclerosis in separate vascular beds which may have important consequences when considering management and treatment of this disease.

## Background

Atherosclerosis is strongly associated with symptomatic cardiovascular disease and ischemic stroke, which are the leading causes of death and disability in the Western world. Atherosclerosis is considered to be a multifactorial disease with numerous risk factors including smoking, alcohol abuse, hypertension, diabetes mellitus, dyslipidemia and infection with microorganisms including *Chlamydia pneumoniae *[1]. All these factors involve complex interactions between pathways associated with inflammation, lipid metabolism, coagulation, hypoxia, apoptosis and the immune response. Atherosclerotic plaque instability is also an independent risk factor for ischemic stroke [2-5]. In the absence of atherosclerosis, normal vessel walls have a microvasculature that is confined to the adventitia and outer media [6]. Intimal neovascularization of human arteries was first noticed and linked with atherosclerosis and intimal thickening by Koester in 1876 [7]. As the disease progresses, the intima thickens and oxygen diffusion is impaired [8]. In atherosclerotic plaques angiogenesis allows the formation of new microvessels to maintain oxygen and nutrient supply for vascular cells [5]. Neovessel growth occurs in regions of atherosclerotic lesions undergoing remodelling, plaque 'shoulders' prone to rupture. **There is a cellular inflammatory response to injury in the early remodelling stage of repair, in which angiogenesis may also play an important role**. In pathological conditions, neovascularization varies from a transient contribution to healing to a permanent contribution for tissue regeneration [9].

A variety of studies, suggest that neovascularization contributes to the growth of atherosclerotic lesions, is associated with symptomatic carotid disease, and is a key factor in plaque de-stabilization leading to rupture [10,11]. Some of the neovessels are irregular and immature, similar to those found in solid tumour vascularization and therefore may contribute to the development of intraplaque haemorrhage and subsequently plaque instability [5,7-9,12].

CD105 is a homodimeric integral membrane glycoprotein composed of disulfide-linked subunits of 90-95 kDa [13] and is a component of the transforming growth factor-β receptor complex. CD105 is predominantly expressed in angiogenic endothelial cells and is up-regulated during hypoxia via the hypoxia-inducible factor-1α which directly binds to the hypoxia response element in the CD105 promoter [14,15]. CD105 is a sensitive marker for identification of tumour neovascularization, growth and prediction of outcome [16,17]. CD105 is also sensitive marker and much more specific than CD31 or TGF-β1 for assessing neovascularization in atherosclerotic plaques [18,19], whilst levels of circulating soluble CD105 can accurately predict the presence of unstable plaques and even plaque rupture [20].

Here, we visualised CD105-positive vessels to ascertain the density of neovessels and the different vessel morphology within the neointima's of large vessel (carotid) and smaller calibre coronary and middle cerebral artery plaques in order to estimate the role of angiogenesis in different stages of development of atherosclerotic lesion from different vascular beds with known histological differences.

## Methods

### Carotid and coronary specimens and anatomo-pathology

We included 38 human carotid arteries with low to moderate stenosis (less than 50% by EcoDoppler imaging, angio-RM or angio-TAC), 20 MCAs and 32 coronary arteries obtained as vascular transplants from organ donors and post-mortem autopsies, Table 1. Carotid arteries, including the common carotid artery, and a large portion of internal and external carotids were excised by a vascular surgeon as a part of a standard procedure for organ transplantation. There was no time delay as the other organs were removed simultaneously. The arteries were removed, immediately rinsed in sterile 0.9% saline, snap-frozen in liquid nitrogen and stored at -80°C.

**Table 1:** Patient's clinical data

***Feature***	***Carotids (n = 38)***	***MCA (n = 20)***	***Coronaries (n = 32)***	***P-value***
Age (Mean ± SD)	67 ± 15*	69 ± 15^†^	52.3 ± 10.8*^†^	0.000
Sex (♂/♀)	25/13	14/6	25/6	NS
WBC × 10^9^/L (Mean ± SD)	12.3 ± 10.0	10.9 ± 6.1	7.6 ± 2.1	NS
Glucose (Mean ± SD) (mmol/l)	6.7 ± 2.0	9,1 ± 5,4^†^	5.7 ± 0.9^†^	0.026
Total cholesterol (Mean ± SD) (mmol/l)	3.4 ± 1.0*	4.6 ± 1.7	5.3 ± 2.0*	0.003
HTA n, (%)	15 (39.5)	13 (65)	5 (16.1)	0.001
DM n, (%)	8 (21.1)	6 (30)	4 (12.9)	NS
DLP n, (%)	9 (23.7)	6 (30)	15 (48.4)	NS
Smoking n, (%)	9 (23.7)	11 (55)	23 (74.2)	0.000
Alcohol abuse n, (%)	8 (21.1)	5 (25)	14 (45.2)	NS
CAD n, (%)	8 (22.2)	5 (25)	10 (32.3)	NS
Statins n, (%)	5 (20.8)	2 (10)	9 (29)	NS
Antiplatelets n, (%)	4 (16.7)	6 (30)	4 (12.9)	NS
Rasb n, (%)	9 (37.5)	6 (30)	12 (38.7)	NS

MCA, middle cerebral arteries; WBC, white blood cells; HTA, hypertension; DM, diabetes mellitus; DLP, dyslipemia; CAD, coronary artery disease; Rasb, renin angiotensin system blockers;

*^† ^Statistic significant between these groups; NS, not significant

Prior to experimentation the arteries were fixed for 24 hours in buffered formalin, briefly decalcified to remove excess calcium and embedded in paraffin. Sections (5 μm) were cut on a microtome. Plaque morphology was evaluated by analysis of haematoxylin-eosin stained serial sections obtained at intervals of 3 mm. Carotid, coronaries and MCA plaques were classified according to the American Heart Association (AHA) [21], with some modifications in the case of carotid arteries because points of calcification are common in normal carotid arteries. Representative sections were used for immunohistochemistry. The study was approved by the local ethical committee in accordance with institutional guidelines and the family's written informed consent was obtained.

### Immunohistochemical analysis

Paraffin-processed sections (5 μm) were deparaffinized in xylene and rehydrated in graded ethanol solutions. Slides were then rinsed in distilled water and treated with 10% hydrogen peroxide in methanol (30 minutes at RT) to remove endogenous peroxidase activity. Sections were blocked with a 5% of normal serum in PBS-tween 0.1% (30 minutes at RT). Slides with the arterial specimens were then incubated with the specified dilution of primary antibody anti-CD105 (1:50, goat polyclonal antibody, R&D Systems, Abingdon, Oxford, UK) or anti-CD34 (1:50, mouse monoclonal antibody, NovoCastra, Newcastle, UK) overnight at 4°C. After washing 3 times in PBS, biotinylated secondary antibody (Vector Laboratories) was used at a 1:200 dilution, and incubated at RT for 1 hour. After rinsing in PBS, standard Vectastain (ABC) avidin-biotin peroxidase complex (Vector Laboratories) was applied, and the slides were incubated at RT for 30 minutes. Colour was developed using 3, 3'- diaminobenzidine (DAB) and sections were counterstained with haematoxylin before dehydration, clearing, and mounting. Negative controls in which the primary antibody was replaced with PBS were used to test for non-specific binding (data not included). All immunostaining was assessed by 2 investigators simultaneously using a double-headed light microscope. Results are presented in Tables 2, 3 and 4.

**Table 2:** N° neovessels/mm^2 ^stained with anti-CD105 compared with anti-CD34 in selected cases

	***CD34*** ***N°neovessels/mm***^2^	***CD105*** ***N°neovessels/mm***^2^
Carotids (n = 4)	7.9 ± 5.2	1.6 ± 1.1
Coronaries (n = 4)	8.8 ± 6.6	2.1 ± 1.6

Values are mean ± SD

**Table 3:** N° neovessels/mm^2 ^in the neointima and Maximum thickness of neointima (μ*m*) of different arteries

	***N° neovessels/mm***^2^	***Neointima thickness (μm)***
***Carotid AHA classification***		
Early (I-III) n = 21	0.04 ± 0.2	626.2 ± 876.5
Intermediate(IV-Va) n = 17	1.8 ± 2.4	1925.7 ± 882.5
***P-value***	0.000	0.000
***Coronaries AHA classification***		
Early (I-III) n = 11	0.0	512.6 ± 327.6
Intermediate (IV-Va) n = 11	1.3 ± 1.9	1141.9 ± 458.8
Advanced (Vb-VI) n = 10	5.9 ± 7.4	1561.7 ± 1433.6
***P-value***	0.000	0.003

***MCA***		
Early (I-III) n = 12	0.0	129.2 ± 176.7
Intermediate (IV-Va) n = 8	0.2 ± 0.4	841.67 ± 455.5
***P-value***	NS	0.001

Values are mean ± SD; MCA, middle cerebral arteries; NS, not significant

**Table 4:** N° neovessels/mm^2 ^in the adventitia of different arteries

***AHA classification***	***N° neovessels/mm***^2^ ***plaque area***	***N° neovessels/mm***^2^ ***non-plaque area***	***p-value***
Carotids			
Early (I-III) n = 21	2.9 ± 3.2	2.4 ± 2.4	NS
Intermediate(IV-Va) n = 17	3.0 ± 2.1	2.1 ± 2.2	NS
Coronaries			
Early (I-III) n = 11	3.7 ± 4.4	1.7 ± 2.5	NS
Intermediate (IV-Va) n = 11	2.3 ± 1.6	0.8 ± 1.3	0.003
Advanced (Vb-VI) n = 10	3.0 ± 2.8	2.0 ± 1.9	NS

Values are mean ± SD; NS, not significant

### Quantitative analysis

Manual microvessel counting was performed by a single observer. All neovessels of the neointimal and adventitial areas were counted using fields at × 200 magnification with the Olympus Vanox AHBT3 microscope in all type of arteries. The density of neovessels of the adventitial layer containing plaque was compared to the non-containing plaque area. Each microvessel was defined as a lumen surrounded by a layer of endothelial cells highlighted by immunostaining with anti-CD34 antibody and each active neovessel was defined as positive with anti-CD105 antibody. The total microvessel count for each sample section was divided by the area covered by that section. Microvessel counts were expressed as microvessels per square millimetre of plaque sectional area. The morphometric study was performed using a Sony DXC-S500 camera and processing performed using the Visilog 4.1.5 (Noesis) program.

In this study, we compared the number of anti-CD105 and anti-CD34 positive neovessels per square millimetre in the neointima of selected carotids (n = 4) and coronaries (n = 4) to confirm the selectivity of the antibodies. The number of microvessesls stained with anti-CD34 antibody was higher than anti-CD105 ones in both types of arteries. Results are presented in Table 2. Thirty eight carotids, 32 coronaries and 20 MCAs were subsequently analyzed for the expression of CD105.

### Statistical analysis

All statistical analysis was performed with the SPSS for Windows statistical software program (version 15.0, SPSS Inc.). One-way ANOVA was used to assess the statistical significance of patient's clinical data and differences between the three groups of arteries in relation to quantitative variables. The Chi-Square test was used for measurement of differences in qualitative variables. Differences in the number of neovessels in the intimas between carotid and coronary arteries was analysed using the Wilcoxon non-parametric test. Differences in the number of neovessels per square millimetre in the neointima and the type of lesion or the maximum thickness of neointima (μm) were analysed with the Kruskal-Wallis test. Means between numbers of neovessels in different areas of the adventitia were compared using the Student-t test. Statistical significance was assumed when *P *< 0.05.

## Results

A summary of the patient's basic clinical data, derived from samples which were obtained before death, is presented in Table 1. Haematoxylin and eosin sections were classified using the AHA system as; type I, early lesion; II, fatty streaks; III, intermediate lesion; IV, atheroma; Va, fibroatheroma; Vb, calcific lesion; Vc, fibrotic lesion and VI, complicated lesion [21]. Plaque AHA classification and phenotype characteristics such as inflammation or calcification are presented in Additional file 1.

### Neointimal plaque vascularity

Of the 38 carotid and 32 coronary arteries, we compared the number of anti-CD105 and anti-CD34 positive neovessels per square millimetre in the neointima of selected carotids (n = 4) and coronaries (n = 4). The number of microvessesls stained with anti-CD34 antibody was higher than anti-CD105 ones in both types of arteries. Results are presented in Table 2.

In the 38 carotids, 32 coronaries and 20 MCAs analyzed for CD105 expression, there was an increase of the maximum thickness of the neointima (μm) in more advanced lesions compared with intermediate and/or early lesions (p < 0.05). There was a significant increase in plaque microvessel density with increased lesion severity in coronary arterial plaques and in carotid arteries (p < 0.05). There were significantly more microvessels per square millimetre in both intermediate and advanced lesions compared with early lesions. Results are presented in Table 3. Few neovessels were observed in the neointima's of intermediate MCAs lesions; however intense expression of CD105 was seen in the intraluminal endothelial cells in all lesion types.

#### Expression of CD105 positive vessels in carotid lesions

**Figure 1:**
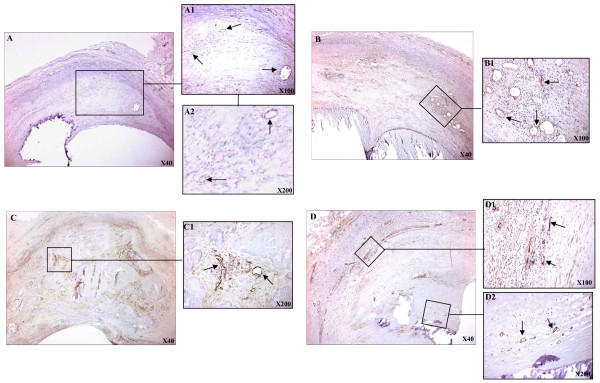
**Representative positive CD105 immunostainig in carotid lesions**. A, neovessels within the lipidic core in a type III/IV plaque. B, An inflammatory area with multiple vessels in a fibroatheroma (arrows). C, Shows a calcified area with microvessels (arrows). D, Shows a fibroatheroma lesion; D1, surrounding lipidic core area; D2, area within the lipidic core. (Magnifications as specified within the figures).

Positive CD105 immunostaining was observed in the few neovessels that there were within and surrounding the lipidic core in type III/IV lesions. The majority of neovessels were circular and regular in shape within the lipidic core (Figure 1A). However, there were also highly irregular, multilobular, partially collapsed vessels surrounding the lipidic core. In fibroatheromatous (Va) lesions there were more highly irregular, multilobular, partially collapsed vessels than regular-shaped circular vessels within inflammatory areas (Figure 1B). The shape of neovessels surrounding the lipidic core (Figure 1-D1), within plaque shoulders, and within calcified areas (Figure 1C) were highly irregular, multilobular, and often, partially collapsed. However, flattened and elongated but patent vessels were also observed within lipidic cores (Figure 1-D2).

#### Analysis of CD105-positive vessels in coronary lesions obtained at transplant or following sudden death

**Figure 2:**
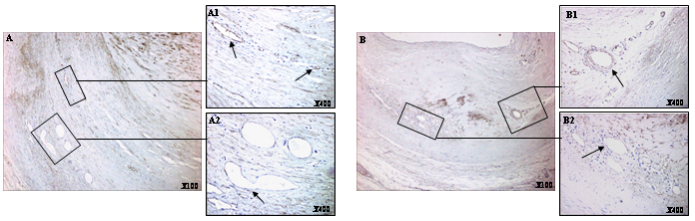
**CD105 noevessel expression in representative coronary arterial plaques**. A, neovessels surrounding the lipidic core in type III/IV lesions (arrows); B, more advanced lesions; B1, the shoulder area of the plaque; B2, internal inflammatory region (arrows). (Magnifications as specified within the Figures).

**Figure 3:**
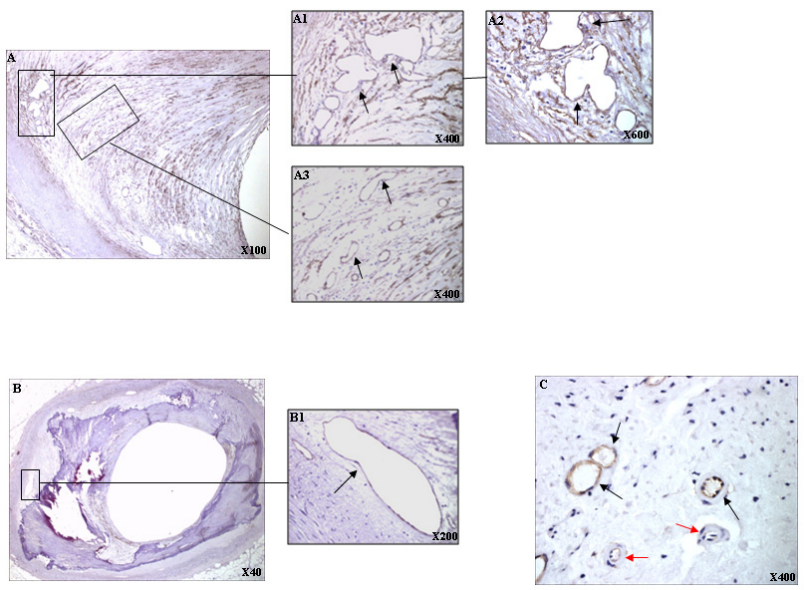
**CD105 noevessel expression in representative advanced coronary arterial plaques; **A1, the area surrounding the lipidic core, and A2, higher magnification showing an example of a multi-lobular abnormally formed neovessel (arrow); A3, shows an area within the lipidic core (arrows). B, shows a calcified area containing vessels (arrows). C, shows immature CD105-positively stained neointimal thin walled vessels (black arrows) adjacent to more mature thicker walled vessels expressing CD105 more weakly or not at all (red arrows). (Magnifications as specified within the Figures).

There were fewer microvessels in intermediate and atheromatous lesions than in advanced lesions. CD105 expression was strongest in the advanced lesions. CD105 immunostaining in the neovessels surrounding lipidic cores of type III/IV lesions were positive. The majority of neovessels were flattened and elongated (Figure 2A). There was an increase in the number of CD105-positive vessels in fibroatheromatous lesions. There were irregular and multilobular vessels surrounding the lipidic core (Figure 3-A1 and 3A2). However, flattened and elongated but patent CD105-positive vessels were also observed within the lipidic core (Figure 3-A3). Within the shoulder different vessel shapes were present: regular and circular vessels and flattened, elongated but patent vessels (Figure 2-B1). There were few inflammatory areas in complicated coronary lesions. Flattened and elongated vessels were present in these areas and in the shoulder (Figure 2-B2). Vessels surrounding calcified lesions were unequivocally positive for CD105. In general there were flattened and elongated but patent vessels in these areas (Figure 3B). In these samples, the majority of the CD105-positive vessels were immature with thin vessel walls and no evidence of smooth muscle cell covering (Figure 3C black arrows). More mature vessels stained weakly or negative for CD105 (Figure 3C, red arrows).

#### Middle cerebral arteries

**Figure 4:**
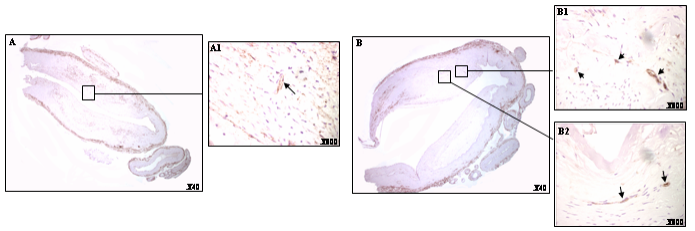
**Positive CD105 immunostaining in MCA**. A, CD105 neovessels in representative atheroma lesion (arrows). B, CD105 positive neovessels in a representative fibroatheroma lesion. (Magnifications as specified within the Figures).

Few CD105 positive neovessels were identified in intimal lesions of these arteries. Only two cases of intermediate lesions demonstrated angiogenesis. These neovessels were small and elongated (Figure 4).

### Adventitial vasa vasorum concentration in plaque containing and plaque free regions

The median total microvessel count per square millimetre in adventitia where there was visible intraluminal plaque did not differ significantly with that of plaque free regions in early or intermediate stage carotid arteries. However there was a significant correlation between CD105-positive *vasa vasorum *in advanced plaque regions of coronary arteries (p = 0.003). Results are presented in Table 4.

## Discussion

In this study, we have identified significantly increased CD105-positive microvessel density with increasing grade of atherosclerotic lesion in carotid arteries with stenosis <50% and in coronary arteries. Increased CD105-positive intimal neovessels were demonstrated previously by Li et al [18], whilst they also showed that CD105 staining was almost absent in normal arteries. Since we previously showed that cells (myoblasts) which do not normally express CD105, when transfected with this gene, exhibited increased cellular attachment, spreading, survival and growth concomitant with increased phosphorylation of JNK [15], and that CD105 expression is also up-regulated by hypoxia and TGF-β, also features of plaque development [22], the current observations suggest that CD105 expression is associated with active angiogenic neovascularization in these lesions. In selected cases we compared CD105 staining with CD34 positive vessels. There was less CD105 positive vessels, further suggesting that this marker is selective for active or new vessels.

There was a statistically significant increase in the maximum thickness of the neointima with increasing grade of lesion in coronary, carotid and MCA arteries. This increase correlated with an increase in the expression of CD105-positive microvessels. The early stage (grade I-III) coronary arteries generally contained stable uncomplicated plaques with few areas of inflammation. This was in contrast to the larger calibre carotid arteries which exhibited more complex heterogeneous features with more evidence of inflammation and the presence of CD105-positive vessels in grade I-III + lesions. This adds more weight to the evidence suggesting significantly different developmental profiles between carotid and coronary artery diseases with that of the carotid arteries demonstrating higher angiogenic activity, possibly in part due to the increased inflammatory infiltration and ultimately resulting in earlier production of unstable regions prone to haemorrhage and rupture [1,12]. The prevalence of neovascularization in carotid arteries of symptomatic patients could help to explain the more rapid development of unstable lesion and subsequent thrombosis. Further, we know from the clinical experience that patients with low to moderate carotid stenosis suffer strokes or transient ischaemic attacks. These often are classified as of unknown origin if screening for cardiac source of emboli is negative and only non-significant intracranial or extracranial stenosis is detected [23,24]. Quickly evolving carotid lesions without important lumen occlusion may be at least partially responsible for these events.

In more advanced plaques (grade IV-V) there remained a notable difference between coronary and carotid vessels with respect to the numbers of neointimal CD105-positive microvessels and this was associated with larger intimal thickness in the carotid arteries. There were no notable differences in plaque CD105-positive vessel density in coronary or carotid arteries from patients who had been undergoing treatment with antiplatelets and statins and the others.

Atherosclerosis in the intracranial arteries is less studied, although stenosis of MCA is a common cause of stroke in some populations [25]. The exact frequency and role of intracranial artery plaques in living patients with stroke is still unknown in Caucassian population [26]. Intracranial plaques and stenoses is highly prevalent in fatal stroke, however, some of the reported stenoses graded 30% to 75% may be causal and correspond to superimposed clot on ulcerated plaque. In our series MCA showed evidence of intimal angiogenesis. This is in contrast to some of the studies which reported higher prevelance of neovasculature in plaques associated with stroke [25]. However, the authors did not use any marker to show neovessels and overall number of plaques with neovasculature was low. Plaques developing in the intracranial arteries can probably survive and obtain nutrients by diffusion from the existing adventitia. However the existence of CD105-positive *vasa vasorum *allows for the possibility that plaque development still activates these vessels and that they are affected by secretion of pro-angiogenic molecules from the intimal site. It would be interesting to compare and identify the mitogenic factors expressed in different vascular beds.

Our results indicate that intraplaque biomarkers, rather than conventional risk factors, may reveal clinical and radiological differences between patients with carotid and MCA atherosclerosis. Plaques associated with MCA atherosclerosis may be more stable than those associated with carotid atherosclerosis [27].

Perhaps surprisingly, there were no significant differences between the numbers of CD105-positive vessels in the adventitia adjacent to the plaque area compared with non-plaque containing regions in carotid arteries. Since the number of CD105-positive vessels was increased significantly in all areas of the adventitia, it is possible that a more general activation of the *vasa vasorum *occurs and neointimal infiltrating endothelial cells are derived from a more widespread region. This was demonstrated recently by Coli et al [28], who showed that the number of CD31/CD34 positive *vasa vasorum *microvessels correlated with plaque echolucency, although not with the degree of stenosis. In contrast, the numbers of *vasa vasorum *CD105-positive vessels correlated strongly with areas of plaque remodelling suggesting a more focussed process of remodelling.

In the neointima we observed abnormal morphology in many of the neovessels even in some of the early stage grade II-III carotid lesions. Some of the vessels demonstrated an elongated appearance but remained patent whilst others were more irregular, multilobular and often collapsed and non-functional. These immature vessels, similar to those identified in growing tumours [29] are more prone to rupture and haemorrhage. Dunmore et al [3] showed that dilated, highly irregular multilobular vessels were found almost exclusively in carotid plaques from symptomatic patients and not in asymptomatic ones. The vessels generally lacked smooth muscle cells, were immature and also existed in regions rich in VEGF-containing macrophages. We also showed that the majority of CD105-positive vessels were thin walled and immature suggesting they were undergoing angiogenesis and/or maturation associated with cellular activation.

Endoglin is a component of the TGFβ receptor complex expressed mainly on the surface of endothelial cells. TGFβ has notable effects on cell proliferation, differentiation, adhesion, migration, and extracellular matrix production, as well as other activities [30]. Endoglin binds to members of the TGFβ superfamily and modulates cellular responses to TGF-β1. The growth factor TGF-β1 is involved in the pathogenesis of atherosclerosis through its pleiotropic effects [31]. Some reports show anti-atherogenic properties to TGF-β1 [32,33], whereas others show that it has atherogenic properties [34,35]. Piao *et al *found an increased expression of TGF-β1 in atherosclerotic lesions and an especially strong over-expression and higher expression rate in the advanced lesions, suggesting that TGF-β1 participates in the formation of early lesions and the progression to advanced lesions [22]. CD105 may initiate its effects in atherosclerosis at an early stage through interaction with TGFβ, but we do not know if the expression of these proteins is causative or a result of the atherogenesis.

It could be useful in a therapeutic setting to differentiate perhaps with specific markers and imaging, patent functional and stable vessels from mal-formed, leaky and unstable ones liable to haemorrhage. Future treatment aimed at stabilization of immature vessels could help in the control of developing lesions.

## Competing interests

The authors declare that they have no competing interests.

## Authors' contributions

AL carried out the histology and immunohistochemistry studies, performed the statistical analysis and drafted the manuscript. MS carried out the iquantification studies and drafted the manuscript. MMT carried out the histology studies. OJB carried out the histology and immunohistochemistry studies. LB participated in its design and coordination. JK participated in the design and coordination of the study, and drafted the manuscript. All authors read and approved the final manuscript.

## Supplementary Material

Additional file 1 **Plaque AHA classification and phenotype characteristics**. AHA classification and phenotype characteristics of carotid, coronary and middle cerebral artery plaques. Click here for file
